# Studies of the intermediary metabolism in cultured cells of the insect *Spodoptera frugiperda *using ^13^C- or ^15^N-labelled tracers

**DOI:** 10.1186/1471-2091-6-24

**Published:** 2005-11-14

**Authors:** Petra Adam, Markus Gütlich, Hartmut Oschkinat, Adelbert Bacher, Wolfgang Eisenreich

**Affiliations:** 1 Lehrstuhl für Organische Chemie und Biochemie, Technische Universität München, Lichtenbergstr. 4, D-85747 Garching, Germany; 2 Forschungsinstitut für molekulare Pharmakologie, Robert-Rössle-Str. 10, D-13125 Berlin, Germany

## Abstract

**Background:**

Insect cells can serve as host systems for the recombinant expression of eukaryotic proteins. Using this platform, the controlled expression of ^15^N/^13^C labelled proteins requires the analysis of incorporation paths and rates of isotope-labelled precursors present in the medium into amino acids. For this purpose, *Spodoptera frugiperda *cells were grown in a complex medium containing [U-^13^C_6_]glucose. In a second experiment, cultures of *S. frugiperda *were grown in the presence of ^15^N-phenylalanine.

**Results:**

Quantitative NMR analysis showed incorporation of the proffered [U-^13^C_6_]glucose into the ribose moiety of ribonucleosides (40 – 45%) and into the amino acids, alanine (41%), glutamic acid/glutamine (C-4 and C-5, 30%) and aspartate/asparagine (15%). Other amino acids and the purine ring of nucleosides were not formed from exogenous glucose in significant amounts (> 5%). Prior to the incorporation into protein the proffered ^15^N-phenylalanine lost about 70% of its label by transamination and the labelled compound was not converted into tyrosine to a significant extent.

**Conclusion:**

Growth of *S. frugiperda *cells in the presence of [U-^13^C_6_]glucose is conducive to the fractional labelling of ribonucleosides, alanine, glutamic acid/glutamine and aspartic acid/asparagine. The isotopolog compositions of the ribonucleosides and of alanine indicate considerable recycling of carbohydrate intermediates in the reductive branch of the pentose phosphate pathway. The incorporation of ^15^N-labelled amino acids may be hampered by loss of the ^15^N-label by transamination.

## Background

Cultured insect cells can serve as efficient host systems for the recombinant expression of certain eukaryotic proteins that fail to be expressed in bacterial host systems. Advantageously, insect cells provide eukaryotic type chaperones, permit the expression of long open reading frames, and are not subject to the limitations caused by bacterial codon preferences [1]. Stable isotope labelling is crucial for NMR structure analysis of proteins using multinuclear spectroscopic techniques. Moreover, differential isotope labelling can significantly enhance the scope of various other spectroscopic techniques such as EPR, infrared and Raman spectroscopy for the analysis of proteins. Differential ^15^N labelling of proteins in Baculovirus-infected insect cells has been achieved *via *^15^N-labelled amino acids present in the growth medium [2,3].

This study was designed to explore in some detail the metabolic network of *Spodoptera frugiperda *cells under the specific growth conditions for ^15^N/^13^C labelling. Our data demonstrate that cultured *S. frugiperda *cells grown on commercial culture media rely on exogenous supply rather than on intracellular synthesis of most proteinogenic amino acids, although central metabolic intermediates (i.e., pentose phosphate, oxaloacetate, pyruvate and acetyl-CoA) acquire label from the proffered [U-^13^C_6_]glucose and transamination activity was observed in the experiment with exogenous ^15^N-phenylalanine.

## Results

In order to assess the utilization of glucose for the biosynthesis of amino acids and ribonucleotides, we cultured *S. frugiperda *cells in a complex culture medium supplemented with a mixture of [U-^13^C_6_]glucose and unlabelled glucose at a ratio of 1:99. The cells were harvested, and lipids were removed by solvent extraction. Cellular RNA was hydrolysed by alkali treatment, and the resulting ribonucleotides were isolated and dephosphorylated. The resulting nucleosides were further purified by HPLC. The residue obtained after alkali treatment consisted predominantly of protein and was hydrolyzed by hydrochloric acid. Amino acids were isolated chromatographically from the hydrolysate.

**Figure 1:**
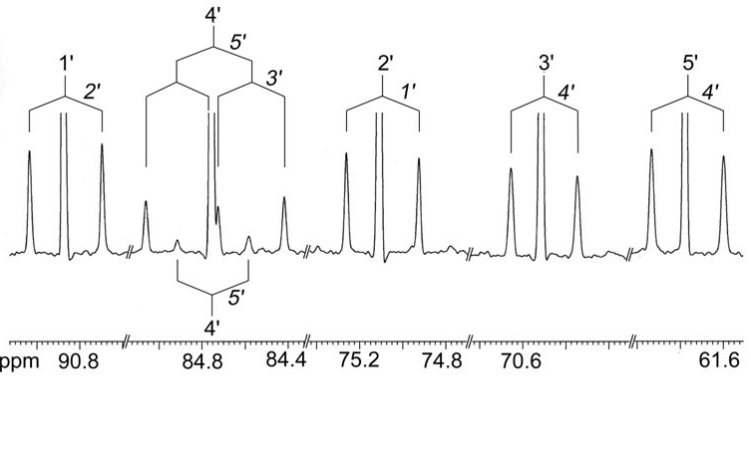
^13^C NMR signals of cytidine from *S. frugiperda *cells grown with a 1:99 mixture of [U-^13^C_6_]glucose and unlabelled glucose added to SF-900 II medium. ^13^C-Coupling patterns are indicated.

^1^H and ^13^C NMR spectra were determined for all isolated metabolites. ^13^C Abundances were determined by quantitative NMR spectroscopy (see Methods). ^13^C Signals of isotopologs carrying two or more adjacent ^13^C atoms were characterized by satellite signals arising by ^13^C^13^C couplings. As an example, the ^13^C signals of the ribose carbon atoms of cytidine showed satellites indicating coupling to one or two adjacent ^13^C atoms (Fig. 1). More specifically, the signals of carbon atoms 1', 2', 3' and 5' appeared as pseudotriplets where the central lines represent molecules with a single ^13^C atom, whereas the satellites indicate molecules with blocks of two ^13^C atoms. The signature of the ribose carbon atom 4' displayed a central line, a doublet and a double-doublet indicating the presence of three different ^13^C-labelled isotopologs, namely [4'-^13^C_1_]-, [4', 5'-^13^C_2_]-, and [3', 4', 5'-^13^C_3_]cytidine, respectively.

**Figure 2:**
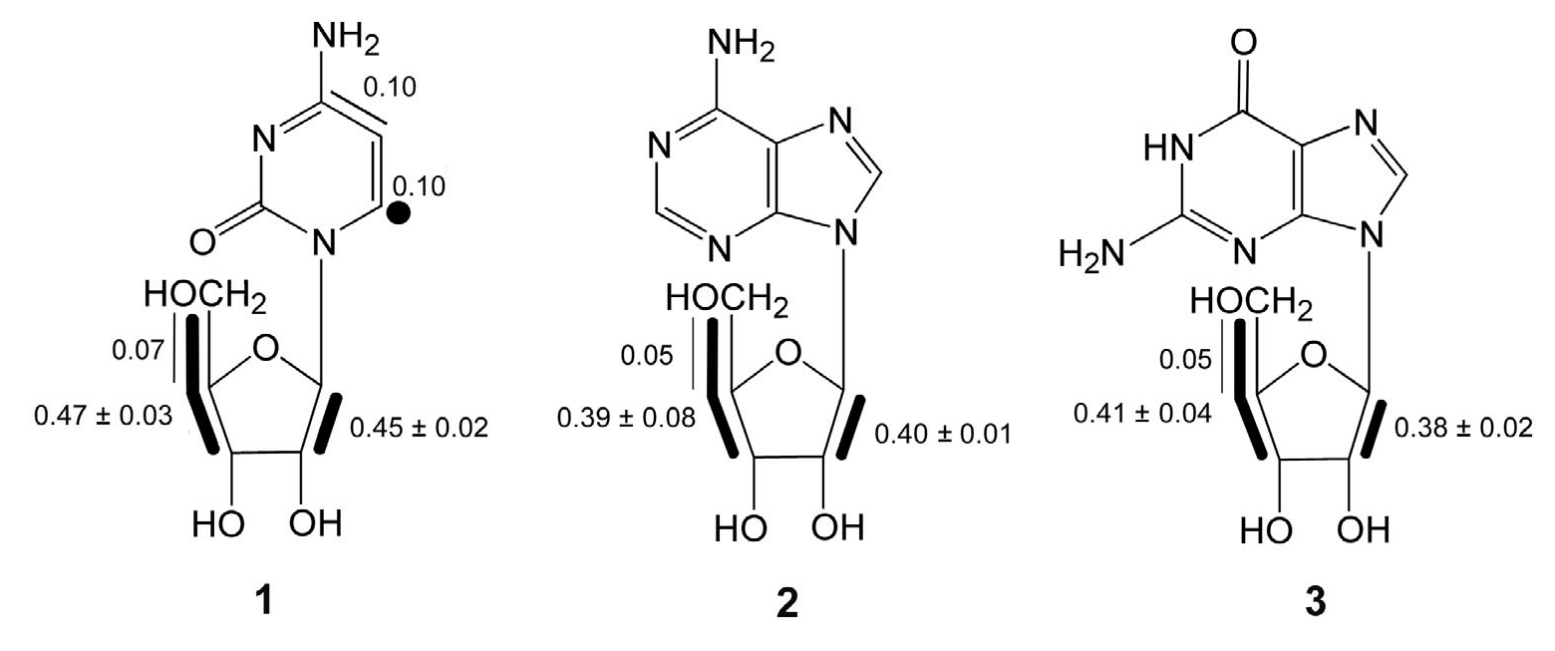
Isotopolog composition of ^13^C-labelled nucleosides from *S. frugiperda *cells grown with a 1:99 mixture of [U-^13^C_6_]glucose and unlabelled glucose added to SF-900 II medium. The filled dot represents a ^13^C_1 _isotopolog detected at an abundance well above the natural abundance contribution. Bold lines indicate isotopologs with adjacent ^13^C-labelled carbon atoms that were transferred from the same molecule of [U-^13^C_6_]glucose. The numbers give the molar enrichments of the indicated isotopologs.

From the relative fractions of each respective satellite pair in the total ^13^C NMR signals (% ^13^C^13^C in Table 1) the abundance (in mol%) of the different isotopologs in the ribose moiety of cytidine (**1**) was calculated (Fig. 2). Notably, the abundance of multiply ^13^C-substituted molecules can be determined independently from the signal pattern of two or more different carbon atoms. The accuracy of the measurements can therefore be assessed statistically. The standard deviations document the accuracy of the method (Fig. 2).

**Table 1:** Isotopolog compositions of nucleosides from S. frugiperda cells grown with a mixture of [U-13C6]glucose and natural abundance glucose.

**Compound**	**Position**	**^13^C NMR Chemical Shift (δ, ppm)**	**Coupling Constants J_CC _(Hz)**	**%^13^C^a^**	**%^13^C^13^C^b^**
Cytidine (**1**)	2	159.7		1.1	
	4	168.4	55.5 (5)	1.3	7.3 (5)
	5	98.9	55.3 (4)	1.2	8.5 (4)
	6	144.4		1.2	
	1'	92.2	42.7 (2')	1.6	22.1 (2')
	2'	76.6	42.5 (1')	1.6	22.8 (1')
	3'	71.9	38.7 (4')	1.5	25.3 (4')
	4'	86.4	38.7 (3'), 42.0 (5')	1.6	23.7 (3', 5'), 2.8 (5')
	5'	63.5	42.0 (4')	1.5	27.4 (4')

Adenosine (**2**)	2	154.7		1.1	
	4	150.6		n.d.	
	5	121.3		n.d.	
	6	157.8		2.7	
	8	142.7		1.1	
	1'	90.4	42.7 (2')	1.7	23.5 (2')
	2'	75.8	42.5 (1')	1.8	22.8 (1')
	3'	72.7	38.5 (4')	1.7	26.0 (4')
	4'	87.9	41.8 (5'), 38.3 (3')	1.6	24.6 (3', 5'), 2.9 (5')
	5'	63.6	41.3 (4')	1.7	25.9 (4')

Guanosine (**3**)	6	156.4		1.3	
	2	153.8		1.1	
	4	150.1		1.1	
	5	115.7		1.0	
	8	135.6		1.2	
	1'	86.5	42.9 (2')	1.7	22.8 (2')
	2'	73.7	42.9 (1')	1.7	22.3 (1')
	3'	70.2	38.4 (4')	1.7	24.1 (4')
	4'	85.2	41.8 (5'), 38.0 (3')	1.6	26.0 (3', 5'), 2.8 (5')
	5'	61.3	42.0 (4')	1.7	26.5 (4')

^a^absolute ^13^C abundance.

^b^relative fraction of satellite pairs in the global ^13^C NMR intensity of the index carbon atom. The coupling partners of the index carbon atom are indicated in parentheses.

**Figure 3:**
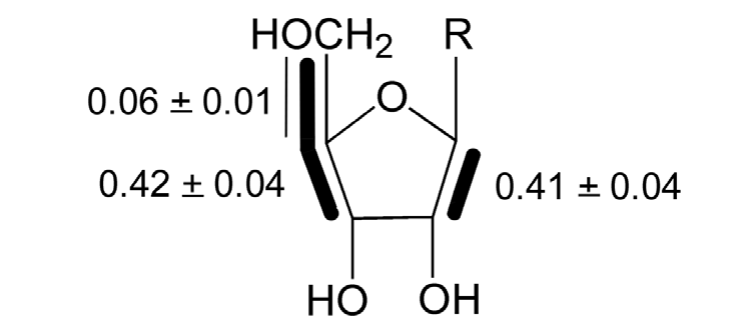
Average isotopolog composition of ^13^C-labelled ribose units in ribonucleosides from *S. frugiperda *cells grown with a 1:99 mixture of [U-^13^C_6_]glucose and unlabelled glucose added to SF-900 II medium. Bold lines indicate isotopologs with adjacent ^13^C-labelled carbon atoms that were transferred from the same molecule of [U-^13^C_6_]glucose. The numbers give the molar contributions of the indicated isotopologs.

The isotopolog compositions of the ribose side chains of adenosine (**2**) and guanosine (**3**) were closely similar to that of cytidine (**1**) (cf. Table 1 and Fig. 2). The sugar units of these nucleosides are all derived from the cellular pentose phosphate pool and the close similarity of their labelling patterns as shown by the low standard deviations (Fig. 3) again document the precision of the experimental data. The average labelling pattern revealed the presence of [1,2-^13^C_2_]- and [3,4,5-^13^C_3_]ribose moieties at relatively high abundances of 0.41 and 0.42 mol%, respectively. [4,5-^13^C_2_]Ribose moieties were present at substantially lower abundance (0.06 mol%). [U-^13^C_5_]Ribose moieties and other multiply ^13^C-labelled ribose isotopologs were virtually absent (less than 0.05 mol%). As described in more detail below, it follows that the ribose pool was primarily alimented by the reductive branch of the pentose phosphate pool, whereas the direct pathway *via *oxidative decarboxylation of the applied [U-^13^C_6_]glucose played an almost negligible role. From the molar fraction of ^13^C-labelled glucose in the medium (approximately 1%) and the molar fractions of multiply ^13^C-labelled isotopologs (see above), a specific incorporation of the proffered [U-^13^C_6_]glucose into the ribosyl moiety of nucleosides can be estimated as 40 – 45% (Table 2).

**Table 2:** Specific incorporation of proffered glucose into metabolites of *S. frugiperda *cells grown in SF-900 II medium.

**Compound**	**Specific incorporation in%**
**Nucleosides**	
Ribose moiety	40–45
Pyrimidine base	10–15
Purine base	< 5
**Amino acids**	
Alanine	45–50
Glutamate/Glutamine (C-4/C-5)	30
Aspartate/Asparagine	10–15
Others	< 5

**Figure 4:**
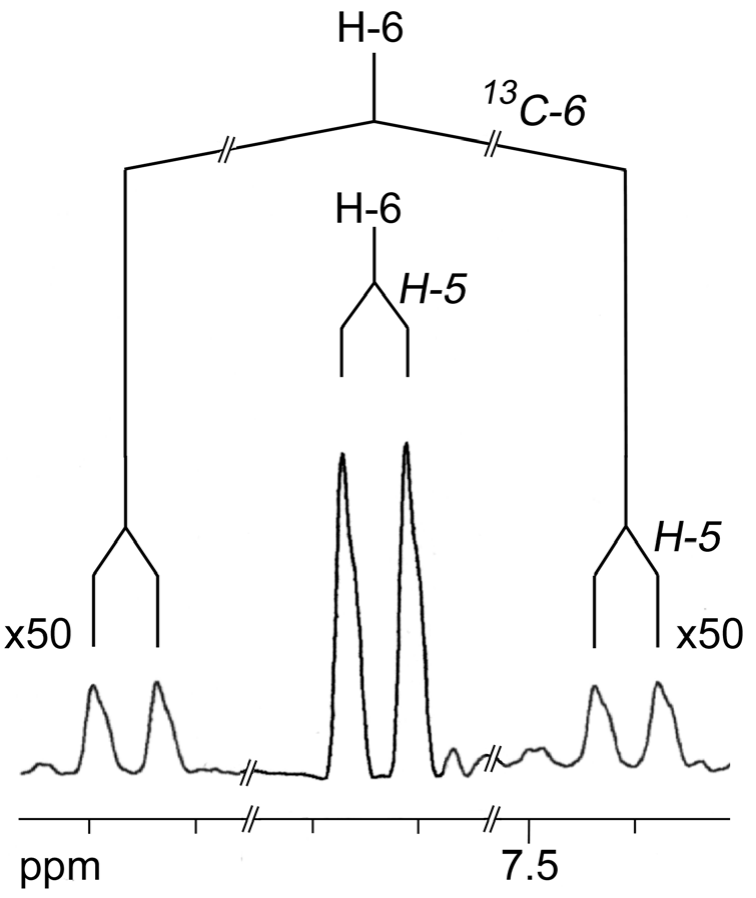
^1^H NMR signal of H-6 of cytidine from *S. frugiperda *cells grown with a 1:99 mixture of [U-^13^C_6_]glucose and unlabelled glucose added to SF-900 II medium. The amplitude of the satellites is 50-fold enlarged by comparison with the central signal. The coupling pattern is indicated.

The ^13^C NMR signals of C-4 and C-5 in the pyrimidine ring of cytidine (**1**) also showed ^13^C^13^C satellites. The quantitative analysis revealed the presence of a [4,5-^13^C_2_]-isotopolog at a molar abundance of 0.1 mol% (Fig. 2) which was significantly above the natural abundance level (0.01 mol%). Moreover, the ^1^H NMR signal of H-6 (Fig. 4) showed ^13^C coupling satellites indicating a ^13^C abundance of 1.2 mol% for the [6-^13^C_1_]-isotopolog, i.e. 0.1 mol% excess over the natural ^13^C abundance of 1.1 mol% (see filled circle in Fig. 2). The presence of these isotopologs demonstrates that a fraction of the pyrimidine was biosynthesised *de novo *from the supplemented ^13^C-labelled glucose *via *aspartate (see below). The specific incorporation of the proffered ^13^C-glucose into the pyrimidine moiety can be estimated as 10–15% (Table 2).

The purine ring systems of adenosine and guanosine showed no significantly increased levels of molecular species carrying two or more ^13^C atoms (Table 1). ^1^H NMR analysis showed no increased ^13^C abundance for the position 8 methine groups of both nucleosides. Consequently, the data demonstrate that purines were obtained from the culture medium and were not biosynthesised to a significant extent (> 5%) under the culture conditions (Table 2).

**Figure 5:**
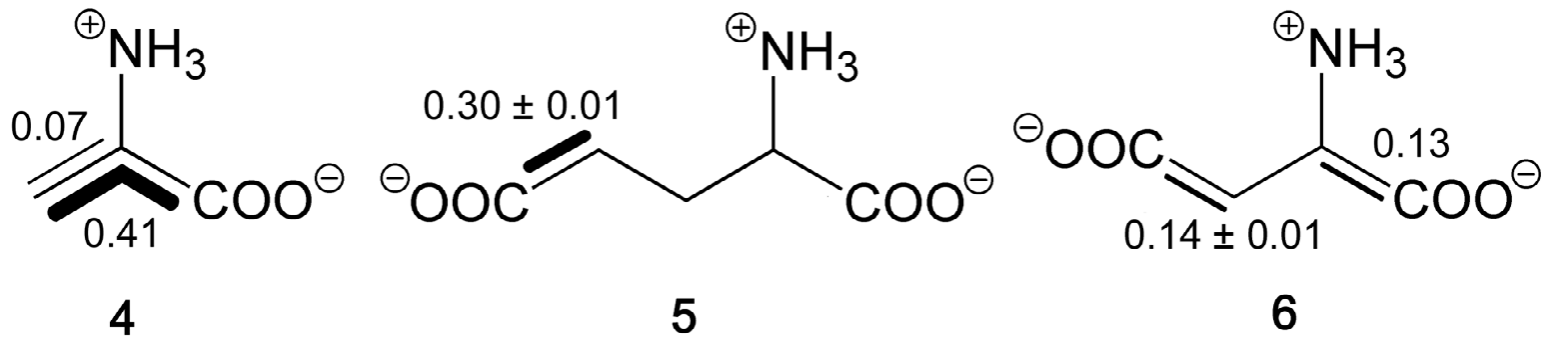
Isotopolog composition of ^13^C-labelled amino acids from *S. frugiperda *cells grown with a 1:99 mixture of [U-^13^C_6_]glucose and unlabelled glucose added to SF-900 II medium. Bold lines indicate isotopologs with adjacent ^13^C-labelled carbon atoms that were transferred from the same molecule of [U-^13^C_6_]glucose. The numbers give the molar contributions of the indicated isotopologs.

Most amino acids obtained from the protein hydrolysate (i.e. leucine, phenylalanine, tyrosine, lysine, histidine, arginine, serine, threonine, valine, proline, methionine and isoleucine) showed only the low intensity ^13^C^13^C coupling satellites typical for natural abundance compounds. In contrast to the spectra of the amino acids mentioned above, the ^13^C NMR signals of alanine (**4**) were characterized by intense satellite signals due to couplings between adjacent ^13^C atoms. More specifically, the signal for C-2 showed a doublet indicating coupling to ^13^C-3, as well as a double-doublet indicating simultaneous coupling to ^13^C-3 and ^13^C-1. The relative fractions of these satellite pairs in the overall signal intensity of the C-2 signal accounted for 3.8 and 22.2%, respectively, which correspond to an abundance of 0.07 and 0.41 mol% for the [2,3-^13^C_2_]- and [U-^13^C_3_]-isotopolog (see also Table 3 and Fig. 5). On this basis, the specific incorporation of the ^13^C-glucose into alanine can be estimated as 45 – 50% (Table 2).

**Table 3:** Isotopolog compositions of amino acids from *S. frugiperda *cells grown in a mixture of [U-^13^C_6_]glucose and natural abundance glucose.

**Compound**	**Position**	**^13 ^Chemical Shift (δ, ppm)**	**Coupling Constants J_CC _(Hz)**	**%^13^C^a^**	**^13^**C**^13^C^b^**
Alanine (**4**)	1	175.7	n.d.	n.d.	n.d.
	2	51.4	59.6 (1), 34.9 (3)	1.8	3.8 (3), 22.2 (1, 3)
	3	18.4	35.1 (2)	1.7	26.5 (2)

Aspartate (**5**)	1	182.9	n.d.	n.d.	n.d.
	2	61.3	60.4 (1)	1.8	7.4 (1)
	3	45.9	55.3 (4)	1.9	7.2 (4)
	4	185.1	55.7 (3)	1.8	7.6 (3)

Glutamate (**6**)	1	176.1		1.3	
	2	56.2		1.0	
	3	27.9		1.1	
	4	32.5	55.1 (5)	1.8	17.1 (5)
	5	179.6	55.1 (4)	1.7	17.2 (4)

^a^absolute ^13^C abundance.

^b^relative fraction of satellite pairs in the global ^13^C NMR intensity of the index carbon atom. The coupling partners of the index carbon atom are indicated in parentheses.

n.d., not determined due to poor signal quality.

Significantly enhanced levels of doubly ^13^C-labelled isotopologs were also found in glutamate (**5**) and aspartate (**6**) (Fig. 5). [4,5-^13^C_2_]Glutamate had a concentration of 0.30 mol% indicating a specific incorporation of approximately 30%. [1,2-^13^C_2_]- and [3,4-^13^C_2_]aspartate were found at abundances of 0.13 and 0.14 mol%, respectively. The abundances of these double-labelled species were closely similar to the abundance of [4,5-^13^C_2_]cytidine (see above).

A second experiment was designed to investigate the incorporation of a ^15^N-labelled amino acid from the culture medium by *S. frugiperda *cells. The standard culture medium for SF-9 cells contains numerous amino acids in relatively large amounts [4]. Moreover, the yeast extract added to the medium contains peptides which may become available to the growing cells after proteolysis. The following experiment was therefore performed with a customized amino acid and sugar free medium which was supplemented with amino acids as indicated in Experimental Procedures. In a preliminary study, we monitored the proliferation rate of cells growing with media containing different amounts of phenylalanine; the addition of 100 mg per litre was found to be sufficient for maximum growth activity.

An incorporation experiment was then performed with medium containing 100 mg of [^15^N]phenylalanine per litre. All other medium components were present in standard amounts (cf. Experimental Procedures). After two passages, the cells were harvested and subjected to acid hydrolysis. Phenylalanine and tyrosine were isolated chromatographically and were analyzed by ^15^N and ^13^C NMR spectrometry, as well as by mass spectrometry.

The ^15^N NMR spectrum showed a signal at 35.8 ppm which was assigned to [^15^N]phenylalanine by internal standardization. The ^15^N abundance was determined by ^13^C NMR spectroscopy. The ^13^C NMR signals for C-2 and C-3 were accompanied by up-field shifted satellite signals due to ^15^N isotope shifts. The sizes of the isotope shifts for C-2 and C-3 (50.9 ppb and 37.6 ppb, respectively) as well as the ^13^C^15^N coupling constant of 3.7 Hz (^1^J_CN_) were in accordance with published values [5,6]. The relative fractions of the signal intensities of the up-field shifted satellites in the overall ^13^C NMR signal intensities of C-2 and C-3 accounted for 25 ± 2%. This value was confirmed by GC/MS of the N-trifluoroacetyl-n-butylester of phenylalanine [7]. A relative abundance of 30 ± 8% was determined for the ^15^N-labelled fragment (m/z = 216). Tyrosine isolated from cell protein was not ^15^N-labelled.

## Discussion

Intermediary metabolism constitutes a complex network with hundreds to thousands of nodes depending on the genetic complexity of the experimental system. The nodes in the central area of the intermediary metabolic network involving the reciprocal transformation of simple carbohydrates, carboxylic and dicarboxylic acids are typically connected by short links. Notably, the sizes and complexity of networks in different organisms can now be compared in some detail on the basis of whole genome sequence data of more than 100 species. The comprehensive quantitative description of such a network requires, in principle, the quantitative assessment of forward and backward reactions mediated by hundreds to thousands of enzymes under intracellular conditions; studies in cell extracts after disruption of cellular integrity can hardly serve as substitute. However, an *in vivo *approach can be performed with cells or organisms on the basis of perturbation/relaxation analysis of the isotopolog distribution in target metabolites [8-16].

This approach can be briefly summarized as follows. Naturally occurring organic matter is a highly complex mixture of isotopologs comprising all stable isotopes of hydrogen, carbon and nitrogen. The natural abundance of ^13^C is about 1.1%. In the quasi-equilibrium mixture of natural organic material of low molecular weight, the isotopologs carrying more than one ^13^C atom are of low abundance; as an example, the approximate natural abundances of some ^13^C-isotopologs of ribose are shown in Table 4.

**Table 4:** Approximate natural abundance of selected ribose isotopologs carrying ^13^C.

**Isotopolog**	**Abundance [mol%]**
[1-^13^C_1_]	1.1
[2-^13^C_1_]	1.1
[3-^13^C_1_]	1.1
[1,2-^13^C_2_]	0.012
[4,5-^13^C_2_]	0.012
[1,2,3-^13^C_3_]	0.00013
[3,4,5-^13^C_3_]	0.00013
[U-^13^C_5_]	0.000000018

The introduction of a ^13^C-labelled compound into any biological system constitutes a local perturbation of the quasi-equilibrium state of isotopolog distribution in the respective metabolic network. Such a perturbation can spread in the network by way of enzymatic interconversion. The comprehensive analysis of the network-wide metabolic relaxation processes resulting from such an initial perturbation requires, in principle, the analysis of isotopolog compositions at virtually every node of the network at various times after the onset of the perturbation. In metabolic networks of typical prokaryotes and eukaryotes comprising hundreds to thousands of nodes, this is a relatively tall order. However, the task can be simplified quite considerably in light of two considerations. (i) Since metabolic networks are highly crosslinked *via *enzyme-catalyzed reactions, it is sufficient to measure the isotopolog distribution at a relatively small number of nodes. The isotopolog composition at numerous adjacent nodes can then be estimated on the basis of certain well-known enzyme catalyzed reactions. (ii) Following a perturbation of the isotopolog equilibrium, the relaxation process is typically not conducive to a true equilibrium state, since anabolic processes lead to a quasisteady state; only catabolic processes are conducive to actual reequilibration.

In practical terms, primary metabolites such as amino acids and nucleic acid constituents are biosynthetically derived from pools of small molecules by anabolic reactions. These primary metabolites are assembled from simple carbohydrates, carboxylic and dicarboxylic acids which are present in low amounts and are at the same time subject to rapid turnover. On the other hand, the products of primary metabolism (amino acids, nucleic acid components) become embedded into polymers (proteins, nucleic acids) where their turnover rate is relatively low. Thus, they reflect the isotopolog distribution of the central intermediary pools (carbohydrates, carboxylic acids) at the time of their formation. Based on these considerations, the transient label distribution in the central metabolite pools can be easily gleaned from analysis of the monomeric building blocks of proteins and nucleic acids (for review, see [13]).

**Figure 6:**
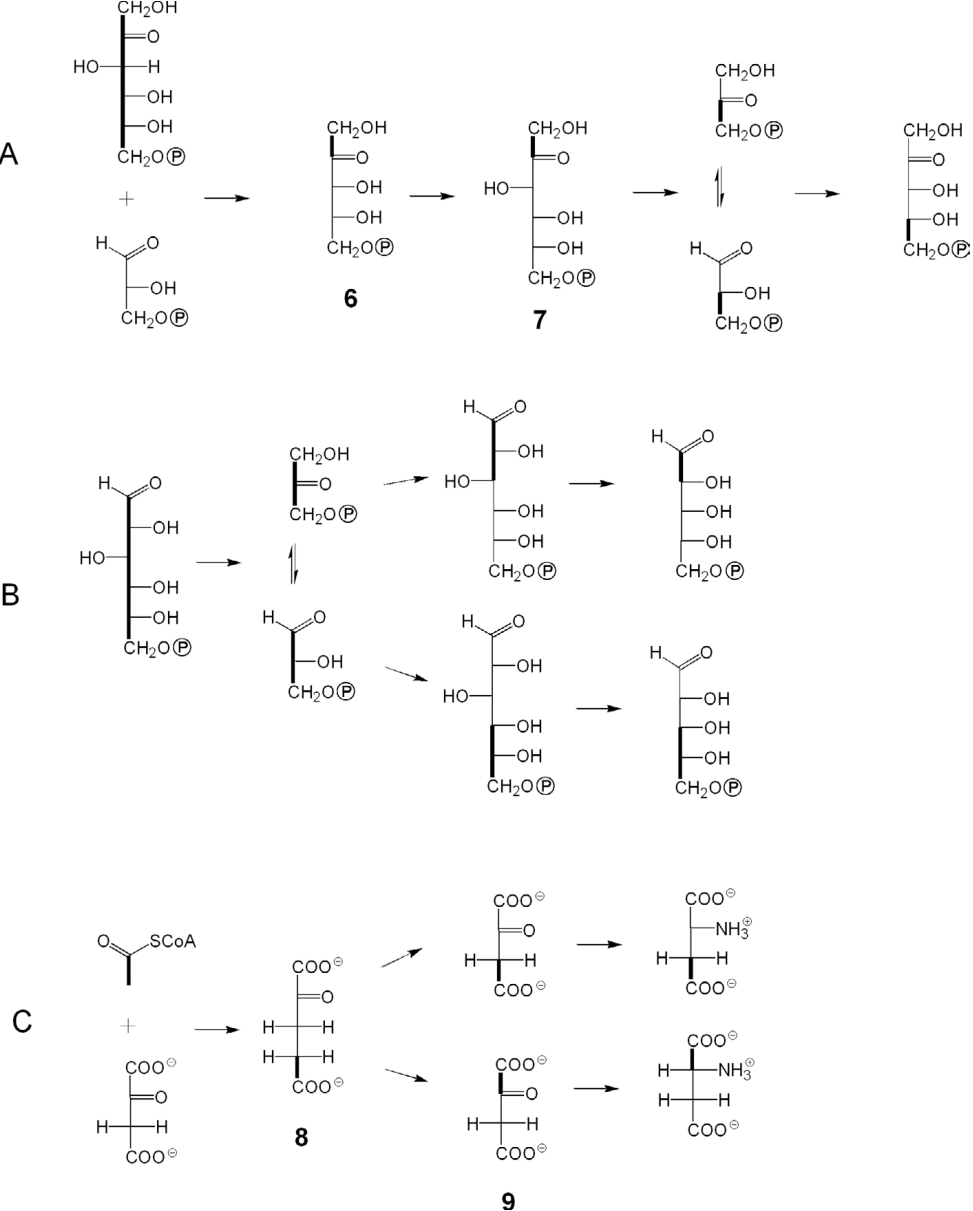
Metabolic processes involved in pentose/pentulose formation from [U-^13^C_6_]glucose in a large excess of unlabelled glucose (A), *via *the pentose phosphate cycle and (B), *via *glycolytic cycling followed by the pentose phosphate cycle. (C), Metabolic processes involved in the formation of the detected aspartate isotopologs. Bold lines connect ^13^C-atoms in a given molecular species.

The formation of pentose derviatives from [U-^13^C_6_]glucose by oxidative decarboxylation *via *the oxidative branch of the pentose phosphate pathway, if active to a significant extent, should have afforded [U-^13^C_5_]ribose phosphate. The virtual absence of that species in the ribonucleosides analyzed indicates that other metabolic processes (i.e. glycolytic cycling prior to any oxidative decarboxylation of glucose) must have played a crucial role. The observed isotopolog pattern can be best explained by cooperation of the reductive branch of the pentose phosphate cycle with glycolysis and regeneration of glucose from triose pool intermediates. Passage through the reductive branch of the pentose phosphate pathway results in breaking of the bond between C-3 and C-4 of glucose. The same is true for glycolysis. Both pathways afford [U-^13^C_3_]glyceraldehyde 3-phosphate from carbon atoms 4 through 6 of the proffered [U-^13^C_6_]glucose. The utilization of that intermediate by transketolase affords a [3,4,5-^13^C_3_]pentulose. On the other hand, grafting of a [^13^C_2_]-fragment derived from [U-^13^C_6_]glucose to an unlabelled glyceraldehyde phosphate moiety affords [1,2-^13^C_2_]pentulose phosphate isotopologs (species **6 **in Fig. 6A).

Glucogenesis using [^13^C_3_]triose phosphates and unlabelled triose phosphates in excess (from the unlabelled glucose fraction) is conducive to the formation of [1,2,3-^13^C_3_]- and [4,5,6-^13^C_3_]hexose phosphates. Oxidative decarboxylation of these species also affords [1,2-^13^C_2_]- and [3,4,5-^13^C_3_]pentose phosphates, respectively (Fig. 6B).

The formation of minor amounts of [4,5-^13^C_2_]pentoses can be explained by the transfer of a [^13^C_2_]fragment from a [1,2-^13^C_2_]pentulose phosphate (species **6**) to unlabelled erythrose phosphate by the action of transketolase. The resulting [1,2-^13^C_2_]fructose phosphate (species **7**) can then be converted to [2,3-^13^C_2_]triose phosphate which affords [4,5-^13^C_2_]pentose phosphate by recycling in the pentose phosphate cycle (Fig. 6A). The labelling patterns of the carbohydrate moieties indicate that ^13^C-label is transferred efficiently to the triose phosphate pool by the joint action of glycolysis and the pentose phosphate cycle.

On the basis of the pyruvate/alanine transamination reaction, the labelling pattern of alanine can be taken as a reference for the labelling pattern of pyruvate. The isotopolog composition of alanine (**4**) was in perfect agreement with the labelling pattern of the three carbon moiety comprising C-3, C-4 and C-5 in the ribose unit of ribonucleosides (cf. Figs. 3 and 5). As discussed above, the latter moiety is biosynthetically equivalent to the triose phosphate pool and, on this basis, the isotopolog compositions of triose phosphate and pyruvate are apparently identical. It can be concluded that pyruvate is predominantly or exclusively biosynthesized from the triose phosphate pool *via *phosphoenolpyruvate and that alternative routes leading to pyruvate (e.g., *via *decarboxylation of oxaloacetate) do not contribute significantly to the *de novo *pyruvate synthesis under the experimental settings.

The presence of [4,5-^13^C_2_]glutamate indicates its formation from [4,5-^13^C_2_]2-ketoglutarate (species **8**) which is assembled from oxaloacetate and [1,2-^13^C_2_]acetyl-CoA *via *the citrate cycle (Fig. 6C). Following the reactions of the citrate cycle, [4,5-^13^C_2_]2-ketoglutarate is converted into a mixture of [1,2-^13^C_2_]- and [3,4-^13^C_2_]oxaloacetate (species **9**) *via *the symmetric intermediates succinate and fumarate. Transamination of these oxaloacetate species yields the detected aspartate isotopologs. Reaction of [3,4-^13^C_2_]oxaloacetate with acetyl-CoA should have afforded [1,2-^13^C_2_]2-ketoglutarate *via *the citrate cycle. Notably, this species is not reflected in the labelling pattern of analyzed glutamate. One possible explanation for this finding is the existence of two compartmented oxaloacetate and/or 2-ketoglutarate pools (i) providing substrates for the citrate cycle and (ii) providing substrates for transamination. Further experiments are necessary to clarify this hypothesis.

By comparison with alanine, the label concentration in the acidic amino acids derived from citrate cycle intermediates is significantly lower. This could be due to the incorporation of exogenously supplied glutamate and aspartate into protein. This is not surprising since the culture medium used contained relatively large amounts of asparagine, aspartate, glutamate and glutamine.

**Figure 7:**
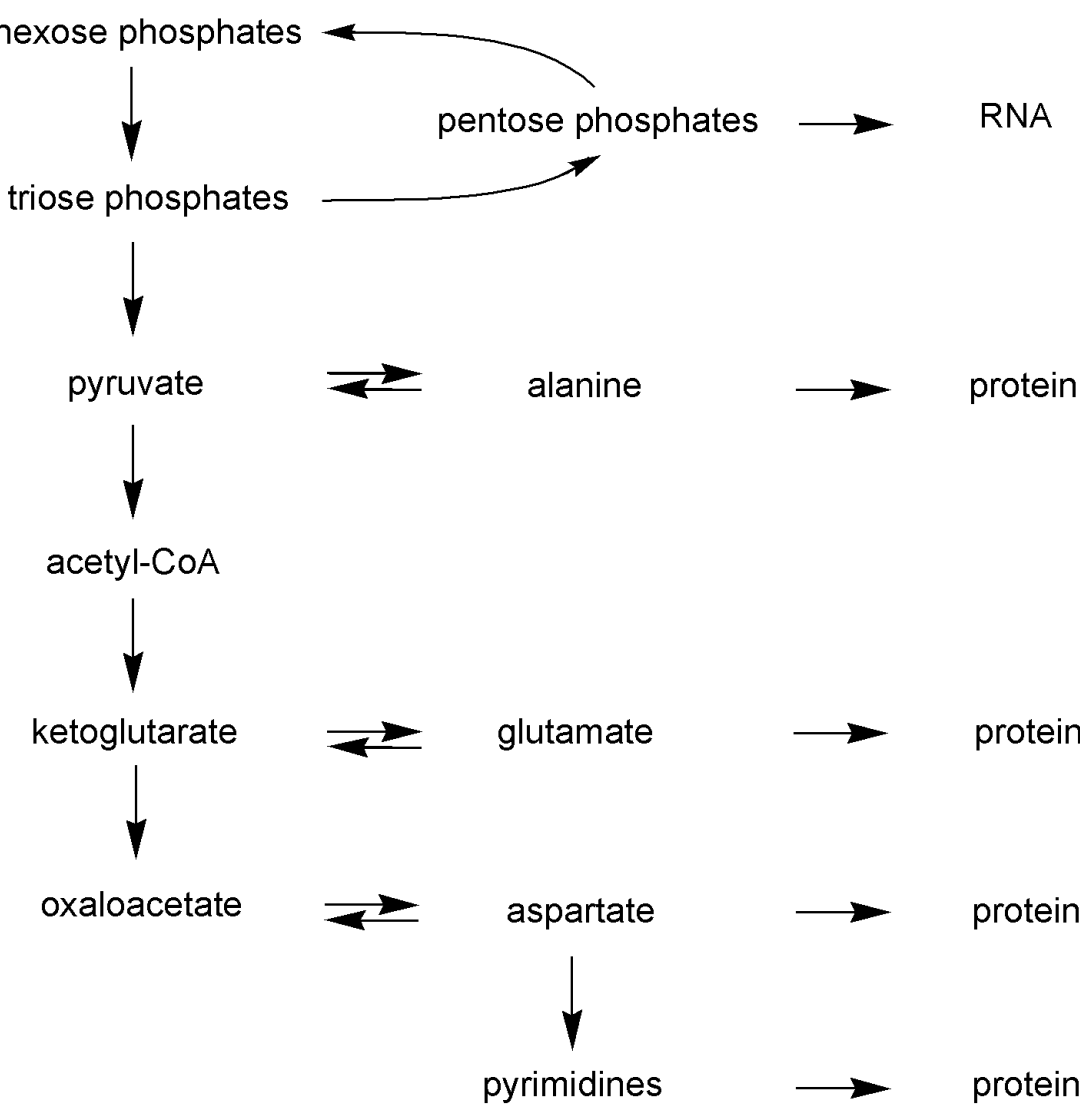
Metabolic network of *S. frugiperda *cells grown in SF-900 II medium.

The virtual absence of label in many amino acids, with the exception of alanine, glutamate and aspartate, indicates that the *de novo *synthesis under the experimental conditions was limited to those amino acids which can be obtained by single step transamination reactions from keto acids which are abundantly present in intermediary metabolism (pyruvate, oxaloacetate and ketoglutarate) (Fig. 7). Under the experimental conditions, remodelling of carbon skeletons for the purpose of amino biosynthesis proceeded at a very low level, if at all.

Recently, the incorporation of ^13^C-glucose into recombinant Abl kinase expressed in Baculovirus-infected insect cells was studied by Strauss et al. [17]. The incorporation rates of glucose into amino acids (i.e., only into alanine, glutamate/glutamine and aspartate/asparagine) were similar to the data presented in our study.

In line with the finding that transamination is the only significant activity with regard to the biosynthesis of aliphatic amino acids, the experiment with [^15^N_1_]phenylalanine shows that transamination occurs on a relatively high level. Although the proffered [^15^N_1_]isotopolog was apparently the only source of phenylalanine, the sample isolated from cell protein retained only about 30% of ^15^N label. This is in notable contrast to an earlier study, where incorporation rates of > 90% have been reported for exogenous ^15^N-phenylalanine into human Abl kinase expressed in Baculovirus-infected insect cells [3,17]. The reasons for this apparent discrapency might be (i) the difference of the protein/protein fraction analyzed by NMR spectroscopy in the two studies, (ii) different concentrations of the supplied ^15^N-phenylalanine, and/or (iii) the difference of the experimental setup with the culture systems.

More specifically, growing cells of the insect were studied in our study. All cellular proteins were then hydrolyzed and amino acids were subjected to NMR analysis. In the studies of Strauss et al. [3,17] insect cells were transfected with Baculovirus and recombinant protein (i.e., c-Abl kinase) was analyzed. The concentrations of exogenous ^15^N-phenylalanine were 10-fold different in the two studies and it cannot be excluded that minor amounts of unlabelled phenylalanine were still present in the culture medium (i.e., "SF-900 II medium without amino acids and without sugar", Gibco) used in our study.

Isolated tyrosine did not contain detectable amounts of ^15^N. This suggests that hydroxylation of phenylalanine does not take place to a significant extent, although phenylalanine hydroxylase is known to be present in insects [18-20].

## Conclusion

Our findings are relevant for the design of protein labelling with recombinant *S. frugiperda *cells. The following aspects need to be considered.

(i) The standardized media contain amino acids in relatively large excess over the real metabolic needs of the cells. Obviously, the concentration of isotope labelled amino acids in the medium should be optimized (i.e., reduced to an appropriate level).

(ii) Differential ^13^C/^15^N labelling of most amino acids (i.e. leucine, phenylalanine, tyrosine, lysine, histidine, arginine, serine, threonine, valine, proline, methionine and isoleucine) can be achieved *via *the respective labelled amino acids present in the growth medium (see also, [2,3,17]). However, labelling with ^15^N could be hampered by the relatively high transamination activity in the cells (cf. also [21]). Hence, the carbon skeleton of specific [^13^C,^15^N]-labelled amino acids would be incorporated intact, but the nitrogen label would be subject to extensive dilution by nitrogen from other, non-labelled amino acids. It may be possible to counteract this isotope loss by the addition of a mixture of [^15^N]-amino acids (e.g., glutamine and asparagine).

(iii) Fractional ^13^C labelled proteins comprising [U-^13^C_3_]alanine, [4,5-^13^C_2_]glutamate, [1,2-^13^C_2_]- and [3,4-^13^C_2_]aspartate can be obtained by addition of [U-^13^C_6_]glucose to the growth medium.

## Methods

### Materials

[U-^13^C_6_]Glucose and [^15^N]phenylalanine were obtained from Isotec, Miamisburg, Ohio, USA. SF-900 II medium was obtained from Gibco, Karlsruhe, Germany.

### Cell culture

SF-9 cells were grown in SF-900 II medium [4]. The cells were kept at a density of 2 × 10^5 ^to 2 × 10^6 ^cells/ml at 28°C in spinner flasks. Expansion cultures were performed in spinner flasks at 28°C and 80 rpm or in a shaking flask at 28°C and 60 rpm. In order to study the metabolic utilization of glucose, the cells were grown in SF-900 II medium supplemented with [U-^13^C_6_]glucose and unlabelled glucose at a ratio of 1:99 (w/w) for two passages.

In order to study the metabolic utilization of exogenous phenylalanine, SF-9 cells were grown in "SF-900 II medium without amino acids and without sugar" (Gibco) with the following supplements: D-glucose (10 g/l), D-maltose (1 g/l), D-sucrose (1.65 g/l), β-alanine (300 mg/l), L-arginine (800 mg/l), L-asparagine (1.3 g/l), L-aspartate (1.3 g/l), L-cystin, sodium salt (150 mg/l), L-glutamate (1.5 g/l), L-glutamine (2 g/l), glycine (200 mg/l), L-histidine (200 mg/l), 2-hydroxyproline (700 mg/l), L-isoleucine (750 mg/l), L-leucine (250 mg/l), L-lysine hydrochloride (700 mg/l), L-methionine (1 g/l), ^15^N-L-phenylalanine (100 mg/l), L-proline (500 mg/l), DL-serine (400 mg/l), L-threonine (200 mg/l), L-tryptophan (100 mg/l), L-tyrosine (disodium salt dihydrate) (360 mg/l), and L-valine (500 mg/l).

### Isolation of amino acids and ribonucleosides

*S. frugiperda *cells were harvested, washed with saline, and lyophilized. The dry powder (3.4 g) was extracted with 42 ml of a methylene chloride/methanol/water mixture (1.1:1:1.1, v/v), as described [22]. The residue was suspended in 14 ml of 1 M NaOH. The mixture was kept at room temperature overnight and was than centrifuged. Nucleotides were isolated from the supernatant as described earlier [23]. Treatment with alkaline phosphatase afforded ribonucleosides which were purified as described earlier [23]. The pellet was hydrolysed by treatment with azeotropic hydrochloric acid for 24 h, and amino acids were isolated chromatographically as described [23]. It should be noted that glutamine and asparagine are converted into glutamate and aspartate, respectively, under the acidic conditions of the hydrolysis. Therefore, analysed glutamic acid and aspartic acid also reflect the contributions of glutamine and asparagine, respectively.

### Derivatization of amino acids

An aliquot of the hydrolysate was lyophilized and dissolved in 200 μl of HCl in n-butanol. The solution was incubated at 100°C for 1 h. Butanol was removed under a stream of nitrogen. The residue was incubated with 50 μl of trifluoroacetic acid anhydride at room temperature. The mixture was evaporated to dryness at 80°C. The residue was dissolved in 500 μl of ethyl acetate. An aliquot was analyzed by gas chromatography/mass spectrometry (column: DB5-MS, J+W scientific, CA, USA; start temperature, 90°C for 3 min; heating rate, 10°C min^-1 ^up to 280°C; 8 min at 280°C).

### NMR spectroscopy

Guanosine was dissolved in 0.5 ml of DMSO-D_6_, adenosine and cytidine were dissolved in 0.5 ml of D_2_O, respectively, and amino acids were dissolved in 0.5 ml of D_2_O/DCl. ^1^H, ^13^C, and ^15^N NMR spectra were acquired with a DRX 500 spectrometer from Bruker Instruments, Karlsruhe, at transmitter frequencies of 500.13, 125.76, and 50.7 MHz, respectively. The data were processed with standard Bruker software (XWINNMR 3.0). Composite pulse decoupling was used for ^13^C NMR measurements. No ^1^H decoupling was applied for ^15^N NMR measurements. All spectra were recorded using a flip angle of 30° and a relaxation delay of 2.0 s. Quadrature detection and quadrature phase cycling were applied in all NMR measurements. The resulting free induction decays were processed by zero filling and multiplication with a Gaussian window function (lb, -1; gb, 0.1). 3-(Trimethylsilyl)-1-propanesulfonate served as an external standard for ^1^H and ^13^C NMR measurements. ^15^N Chemical shifts are reported relative to the ^15^N NMR signal of N-5 of [U-^15^N_4_]6,7-dimethyl-8-ribityllumazine at 327.0 ppm. ^1^H and ^13^C NMR signal assignments were taken from [23].

### Assessment of isotopolog composition

The isotopolog compositions of nucleosides and amino acids were determined by quantitative NMR spectroscopy (Tables 1 and 3) [24]. Relative ^13^C abundances were obtained from signal intensities in one-dimensional ^13^C NMR spectra. Specifically, the signal integrals (I*) were determined for each ^13^C NMR signal of the metabolite under study. Using the same experimental settings, the signal integrals (I_ref_) of the same compound at natural ^13^C abundance were determined. The ratios of the signal integrals of each respective carbon atom (I*/I_ref_) were then calculated. Absolute ^13^C abundances were determined for certain carbon positions from the ^13^C coupling satellites in the ^1^H NMR spectra (for an example, see Fig. 4). Relative ^13^C abundances (I*/I_ref_) were then referenced to the absolute enrichments.

In NMR spectra of multiply labelled samples displaying ^13^C^13^C coupling (for an example, cf. Fig. 1), each satellite signal in the ^13^C NMR spectra was integrated separately. The relative fraction of each respective satellite pair in the total ^13^C NMR signal integral of a given carbon atom was calculated (% ^13^C^13^C in Tables 1 and 3). The relative fractions of satellite pairs were then referenced to the overall ^13^C abundance of a respective carbon atom which was determined as described above. This resulted in molar contributions (mol%) for each respective isotopolog.

## Authors' contributions

PA isolated the metabolites, MG grew the cells, HO and WE were responsible for the NMR analysis, WE and AB drafted the manuscript.
